# The Serious Neglect of Scientific Obstetrics

**Published:** 1907-10

**Authors:** P. W. Van Peyma

**Affiliations:** Buffalo, N. Y.; Associate Professor of Obstetrics, University of Buffalo


### BUFFALO MEDICAL JOURNAL.

VOL. LXIII. OCTOBER, 1907. NO. 3

### ORIGINAL COMMUNICATIONS.

### The Serious Neglect of Scientific Obstetrics. The Remedy

BY P. W. VAN PEYMA, M. D., Buffalo, N. Y.

Associate Professor of Obstetrics, University of Buffalo.

A PAPER with the above title was recently read at a meeting of the Medical Club of this city. The conditions therein pointed out are of the deepest public concern, and particularly, and most directly, affect womankind and the newborn. It has, therefore, been urged that the paper be rewritten, technical expressions eliminated, and copies furnished for distribution.

The purposes of this review are to call attention to the fact that the average standard of obstetric practice is very low; to show that the results are very serious to mothers and infants; and to urge that this condition can and ought to be ameliorated. The truth of these assertions is not disputed by anyone familiar with the facts. One hears of evidence on every side, and the experience of the writer in several hundred obstetric consultations occurring in the private practice of fully ninety physicians, corroborates what is only too apparent. It is well within the truth, to say that of all the departments or specialties of medicine, obstetrics is the one showing the greatest want of intelligence, and the most unskilful and harmful practice. Daily, women and infants are being seriously and needlessly injured. Sometimes the result is the death of the patient or patients; sometimes lifelong suffering and invalidism. But, like many another evil, its very universality seems to result in inattention and tacit acquiescence; and the attitude of a large part of the medical profession and of the community seems to be expressed in the phrase,—Only another necessary evil.

Naturally the question arises—Why is this? The causes which have brought about and which are instrumental in maintaining this unfortunate state are numerous. At best, the practice of obstetrics is wearisome and tedious. It is irregular and time-consuming. One who practises midwifery can count on no time as his own. He is forever tied up, to be ready at a moment's notice; and his nights are often more occupied than his days. Proper attendance upon cases of labor, not hurried to a conclusion, demands much time, and this interferes seriously with other practice. The general practitioner, in attendance upon all classes of diseases, is called upon to take the most special precautions to insure against the transmission of infection to the woman in labor. The practice of obstetrics is, on the average, ridiculously underpaid, reflecting the low estimate of both the medical profession and the laity. The fact that the vast majority of labors are natural, and that delivery in these cases is effected unaided, explains this feeling; as, also, the quite general conviction that no particular knowledge or skill is required on the part of the attendant. And yet, daily, most serious consequences testify to the contrary, since there is scarcely a case where intelligent and skilful attention is not of the greatest value. In very many it means the difference between health and invalidism; and in not a few cases decides the question of life or death of the mother and infant.

There is scarcely anything more important in the management of a case of labor than the conservation of the nervous energy of the patient. The establishment at the very commencement, and the maintenance throughout the whole process of labor, of the right mental attitude on the part of the patient, is a matter of the greatest importance. It determines in many cases whether nature will be able to effect an unaided delivery. This means, in many instances, an early and a long-continued attendance. It means the setting aside of other business. It means overcoming the temptation to apply forceps and get away. It means, in the minds of the laity, less service and, naturally, less emolument. For is it not true that no operation was performed? Probably not even a laceration sewed up. And, yet, it is precisely this treatment that, in the vast majority of cases, means the safety and welfare of the family. Time and nature are great physicians. That the very preponderance of natural, normal cases, through resulting inattention, trusting to luck, and want of careful study, lowers the standard of the practice of obstetrics and, thus, indirectly but positively, increases infant and maternal suffering and mortality, seems paradoxical, but is, nevertheless, most true.

The comparative general observance, during later years, of absolute cleanliness, or asepsis, in the management of cases of labor, has been of infinite value. And yet this, also, has been a two-edged sword. The relative impunity with which surgical procedures can be undertaken nowadays has led to a very great increase of operative interference which, while not, as would formerly have been the case, resulting in infection and perhaps death, yet is responsible for many unnecessary injuries to mothers and newborn. Lacerations, especially, are so common that they seem to be taken almost as a matter of course, and wonder is expressed when they fail to occur. Considering the foregoing, it is not difficult to understand why the practice of obstetrics is in disfavor with, and neglected by, the medical profession, and why the results are so generally bad.

Physicians practising midwifery are divisible into two broad classes,—the general practitioner, and the gynecologist, or specialist in diseases of women. The man in general practice does more or less obstetrics because his families expect it, and because he appreciates the additional income. The gynecologist is called in because his specialty is considered as allied to that of obstetrics. Both, however, consider this practice as relatively undesirable; and, with increasing financial independence, gradually withdraw from it. Except in the very largest cities, specialism in obstetrics is almost unknown. And, yet, the attainment of sound judgment, thorough understanding, and competent skill involves the careful study of many cases, normal as well as abnormal.

Of the circumstances mentioned as militating against safe and satisfactory obstetric practice some may, in the course of time, find their own correction. One, at least,—the tendency to hurry,—will perhaps always remain. It should be kept well in mind that the safe conclusion of the ordinary case of labor requires as much time as in the days two thousand years ago. In our age of rush and hurry, this most important truth is very generally ignored. But, however important the preceding considerations may be, there remains one source of neglect and mischief which undoubtedly outranks in importance all the others. The want of proper, adequate and, especially, clinical or bedside teaching of obstetrics is a matter of reproach, and demands the most urgent and serious consideration.

There is infinitely more to the subject of obstetrics than is generally recognised,—than is recognised by any except those who have given it exact and comprehensive study. That adequate instruction at the bedside is absolutely essential to a proper and practical understanding must be selfevident, even to the laity. To explain fully this importance, in detail, to the lay mind would involve some difficulties. There are, however, certain general facts and principles which even the laity need have no difficulty in grasping.

In the course of delivery the child passes through the bony canal called the pelvis. In the infant the head is the largest and least compressible part. The pelvic canal is not a regularly cylindrical tube, nor is the fetal head a regularly globular mass. Both are irregular in shape, and present various diameters. At the completion of pregnancy, the size of the head approximates the capacity of the pelvis. In order that the passage of the head may be naturally possible it must enter in a certain way and, as it advances, must undergo definite movements of turning and flexion in order, finally, to emerge with the smallest circumference engaging. Failure in any of these particulars means trouble, and may mean positive obstruction. In ninety-seven per cent. of deliveries the fetal head is in advance, and of these cases of head presentation, in four out of five, the infant's back is anteriorly toward the abdomen of the mother. This is the most normal position, the one most favorable for easy and safe delivery. In about one-fifth of all cases, however, the infant's back is turned toward the back of the mother, and these offer delay and various complications. If left to nature, nearly all eventually rotate, so as to bring the back to the front; and delivery is effected naturally, although very generally considerably delayed.

The back of the head is called the occiput; therefore, these varieties are called occiput posterior positions. The other four-fifths, with the back of the head in front, are termed occiput anterior positions. The various natural movements of the child, in passing through the parts of the mother, constitute what is known as the "mechanism of labor." The exact position of the child is usually determined by both an external and an internal examination. The external examination consists in feeling for the parts of the child and in listening for the fetal heart sounds through the abdomen of the mother. The internal examination admits of feeling and locating the membranous lines and spaces existing between the various bones forming the vault of the head. These are called sutures and fontanelles. To recognise and locate these is not an easy matter, and proficiency in this respect requires much practice. Sometimes they cannot be felt distinctly, and it then may become necessary to locate an ear, in order to be sure of the position.

This recognition of the exact position of the head is of absolute importance to scientific obstetric practice. It can only be learned by means of most careful study and teaching at the bedside. The chief sources of bad practice are found in a failure to appreciate the essential importance of the mechanism of labor; in a neglect or inability to determine the exact position of the head; and in impatience and haste in delivery.

Occiput posterior positions occur, as has been said, about once in five cases. For various reasons, the labor in these cases is unusually prolonged. Yet, if left to nature, the outcome, in the vast majority of instances, is a normal delivery. In the practice of the average physician, the result is quite likely to be otherwise; there exists unusual delay, the fact of the occiput being posteriorly is unrecognised or ignored; patient, family and physician are impatient to have the case over with. Forceps are applied, the head is pulled out in the wrong position, and, in nearly every instance, the mother is badly torn, and the infant, also, more or less seriously injured. These cases are safer in the care of a good cleanly midwife than in the hands of the average physician who applies the instrument wrongfully.

Professor Penrose, of Philadelphia, in an article in the American System of Obstetrics, says: "If I were to be asked what one obstetric difficulty, in my experience, had caused most maternal and fetal deaths, what one had caused most maternal and fetal accidents not necessarily fatal,—accidents, however, often making the rest of life worthless, or, still worse than merely worthless, a tragedy,—I think I would say occipito-posterior positions, where the occiput had rotated into the hollow of the sacrum, and which had been improperly treated."

A very large proportion of physicians practising obstetrics, probably at least ninety per cent., ignore the facts that the head and the pelvis are both irregular in shape, and that it requires a proper adjustment of the one to the other to effect a normal delivery. They do not concern themselves with the mechanism of labor, nor with the determination of the position of the head. And the results are what might be expected.

In another class of cases, a want of attention to certain fundamental rules results in equally disastrous consequences. The largest circumference of the head is one drawn around the chin in front, and the back of the top of the head behind. Any circumference drawn from the back of the neck behind to any point of the face in front, is much shorter. If, in the final delivery of the head, the face is allowed to be born while the back of the head is still unborn, a very large circumference engages, and a tear is sure to result. And this is what frequently happens, and explains the occurrence of many tears, entirely unnecessary. The too early, to say nothing of the unskilful application of the forceps; and the too rapid delivery, not giving the tissues time to relax and distend, is a third prolific source of serious tears. Intelligent and careful management, ensuring the engagement of the proper circumference, and allowing sufficient time, will prove how preventable are at least nine-tenths of the tears now daily occurring.

There is a very prevalent and growing notion that in this progressive and enlightened age a woman in labor need only take some chloroform, undergo an artificial delivery and awake, to find all well. And this false conception is fostered by many physicians, either from ignorance or because of a selfish desire to hurry the case, and to be at liberty to attend to other affairs. But this practice is fraught with danger, and the conclusion is always more or less disastrous. To obtain results that will be happy for both mother and infant, time and nature must be given opportunity, and the prospective mother must also do well her part. She must exercise a reasonable amount of patience and of selfcontrol. An appeal must be made to her intelligence, good sense and maternal instinct. The nature of labor, the necessity for time, and the dangers of too early interference should be explained. Also, that a hurried labor is cruel, in that it causes more suffering at the time of delivery, as well as invalidism in the future.

Gynecologists are kept busy with surgical operations, in attempts, more or less successful, to repair the results of bad obstetric practice. If this fruitful source of supply, and one other,—that of infection,—could be removed, there would remain little for gynecologists to do. Would this not be a great boon for womankind—for all humanity? Should not every effort be made to attain it? This would be preventive medicine. Operations are at best but curative, and frequently not even that. Obstetric lacerations, with the resulting scar tissue, predispose to the development of cancer. They are also often a source of serious domestic infelicity. It must be that, when the facts are fully realised, the means for correcting the condition will be forthcoming.

In all cases of labor, and especially in those more or less difficult, it is important to watch the condition of the child. Practically, the only way to determine the actual state of the infant is to listen to the fetal heart sounds. The conditions of the mother and of the child are the circumstances that must decide whether, in a given case, intervention and artificial delivery are demanded. And, yet, this important examination is very much neglected. There are physicians who have practised obstetrics for years and have never heard nor even listened for the fetal heart sounds.

The writer desires simply to prove his contention that the practice of obstetrics is at a very low ebb. He does not wish to arraign the medical profession too severely, nor to unfold a tale of horror. And yet it should be known that mutilated, mentally enfeebled, paralytic, and dead children; lacerated, invalided and dead mothers are the ever recurring product of unintelligent and conscienceless obstetric practice. And this among all classes of society.

A rehearsal of individual cases, seen in the course of more than thirty years of active practice, would, undoubtedly, serve to more deeply impress and fix the points made. It is, however, omitted as perhaps unnecessary, and as unfitted for an article intended for general distribution. It should also be remembered that attention has been directed to only a very limited part of the subject, and that, for the sake of brevity, much has been omitted which would have added to the proof of indifference and neglect. Neither should it be overlooked that all that has been said regarding the want of opportunity to teach obstetrics clinically applies equally to the training of nurses. Some time since a certain nurse, a graduate from one of our larger institutions, was engaged for a case of labor. She was honest enough to say that she had never seen a case. The family being determined to have her, an additional nurse from a distant city was called in to give her some preliminary instruction, and to be present at the confinement.

Can these things be and not excite our special wonder? Can they be and not excite the determination to correct them? It must be, that, once fully realised, womankind, at least, will rise to the occasion, will in selfprotection and in defense of the helpless newborn, insist upon and assure more adequate teaching, more intelligent and conscientious attention.

The plane of development and efficiency to which a graduate has arrived when he leaves his Alma Mater presages, with scarcely an exception, that of his future professional career. The physician who enters upon the practice of his profession with no clear understanding, no practical experience, may possess boldness and assumption, for these are but the indices of ignorance, but he can have no genuine interest in the science, and no adequate skill in the art which he has chosen for his life's work. The result of better training would be to make the practice of obstetrics less disagreeable. At best, it will always remain irksome and exacting. But one learns to enjoy doing that which one does well, and one becomes interested in proportion to one's understanding. To one who has given the subject a fair amount of study, there is scarcely a case that does not present some point of special interest, nor scarcely one that does not teach something additional. The mechanism of labor when well understood and intelligently observed, cannot fail to excite interest and admiration.

Until recently, it was a common experience for medical men to enter upon their private practices without having seen a single case of labor. This is less common now, but still many a man graduates whose experience in this respect is practically nil. It is a fact, also, and one important to remember, that a young medical man will learn more from a half dozen cases carefully studied and fully taught, than he will learn from the first hundred cases seen, here and there, with himself alone in attendance; to say nothing of the mothers and children spared needless injury and suffering.

If we are to have better practical obstetrics, if we are to have fewer mothers and newborn suffering the results of careless, conscienceless and ignorant practice, we must very greatly extend and perfect the opportunities for clinical teaching. At present all the clinical material which this city affords consists of a small number of cases scattered among half a dozen institutions. There is need, most urgent need, for a hospital with sufficient clinical material,—that is, with a sufficient number of cases of labor,—an institution large enough to warrant the residence and the constant supervision of a thoroughly competent teacher. That this object may be realised, an endowment is necessary, to permit of taking cases regardless of ability to pay. It must be evident that the good which an institution of this character could do is incalculable. Such opportunities for practical study exist in many cities in this country and in Europe. The Sloane Maternity of New York City is a splendid example.

Our city was the first in this country, nearly fifty years ago, to introduce the clinical teaching of obstetrics. Public prejudice caused its temporary abandonment. An enlightened public sense has long since caused its reestablishment throughout the civilised world. Who can calculate the suffering, the misery and mortality which, occurring during the many years, must be charged to a blind and foolish prejudice?

So long as the race shall continue, womankind will bear the burden of maternity. Does not simple justice demand that this burden be made as easy and light as conscientious, sympathetic and intelligent care can make it? This article has been written in the hope and in the belief that, the facts being known, something will be done to correct the condition; that somewhere the seed sown will take root and bear fruit; that someone will feel it worth while to make provision for the safeguarding of maternity and of the generations of infants yet unborn.

445 William Street.

---

### ILLUSTRATIONS FOR MEDICAL JOURNALS.

A. J. Martin, (*Medical Record,* March 30, 1907,) discusses the subject of preparing illustrations for periodicals and expresses the opinion that for ordinary use line drawings are more satisfactory than half-tone reproductions from photographs. Examples are given showing the advantages of retouching photographs intended for reproduction.

